# 'Which treatment do you believe you received?' A randomised blinding feasibility trial of spinal manual therapy

**DOI:** 10.1186/s12998-024-00561-0

**Published:** 2025-01-14

**Authors:** Javier Muñoz Laguna, Astrid Kurmann, Léonie Hofstetter, Emanuela Nyantakyi, Julia Braun, Lauren Clack, Heejung Bang, Mazda Farshad, Nadine E. Foster, Milo A. Puhan, Cesar A. Hincapié, the SALuBRITY Blinding Clinician Group, Malin Mühlemann, Curdin Caviezel, Marco Ehrler, Melanie Häusler, Céline Höltschi, Daniela Kroismayr, Serafin Leemann, Daniel Mühlemann, Luana Nyirö, Lorene Rabold, Christof Schmid, Bedran Yilmaz, Fredrik Granelli, Christophe Sem

**Affiliations:** 1 Musculoskeletal Epidemiology Research Group, University of Zurich and Balgrist University Hospital, Zurich, Switzerland; 2 Epidemiology, Biostatistics and Prevention Institute (EBPI), University of Zurich, Zurich, Switzerland; 3 University Spine Centre Zurich (UWZH), Balgrist University Hospital, University of Zurich, Zurich, Switzerland; 4 Institute for Implementation Science in Health Care (IfIS), Medical Faculty, University of Zurich, Zurich, Switzerland; 5 Department of Infectious Diseases and Hospital Epidemiology, University Hospital Zurich, Zurich, Switzerland; 6 Division of Biostatistics, Department of Public Health Sciences, School of Medicine, University of California, Davis, USA; 7 Department of Orthopedics, Balgrist University Hospital, University of Zurich, Zurich, Switzerland; 8 STARS Education and Research Alliance, Surgical Treatment and Rehabilitation Service (STARS), The University of Queensland and Metro North Health, Brisbane, Australia

**Keywords:** Blinding success, Double-blind method, Feasibility study, Back pain, Manual therapy, Masking, Randomised controlled trial, Spinal manipulation

## Abstract

**Background:**

Blinding is essential for mitigating biases in trials of low back pain (LBP). Our main objectives were to assess the feasibility of blinding: (1) participants randomly allocated to active or placebo spinal manual therapy (SMT), and (2) outcome assessors. We also explored blinding by levels of SMT lifetime experience and recent LBP, and factors contributing to beliefs about the assigned intervention.

**Methods:**

A two-parallel-arm, single-centre, placebo-controlled, blinding feasibility trial. Adults were randomised to active SMT (*n* = 40) or placebo SMT (*n* = 41). Participants attended two study visits for their assigned intervention, on average seven days apart. The primary outcome was participant blinding (beliefs about assigned intervention) using the Bang blinding index (BI) at two study visits. The Bang BI is arm-specific, chance-corrected, and ranges from − 1 (all incorrect beliefs) to 1 (all correct beliefs), with 0 indicating equal proportions of correct and incorrect beliefs. Secondary outcomes included factors contributing to beliefs about the assigned intervention.

**Results:**

Of 85 adults screened, 81 participants were randomised (41 [51%] with SMT lifetime experience; 29 [39%] with recent LBP), and 80 (99%) completed follow-up. At study visit 1, 50% of participants in the active SMT arm (Bang BI: 0.50 [95% confidence interval (CI), 0.26 to 0.74]) and 37% in the placebo SMT arm (0.37 [95% CI, 0.10 to 0.63]) had a correct belief about their assigned intervention, beyond chance. At study visit 2, BIs were 0.36 (0.08 to 0.64) and 0.29 (0.01 to 0.57) for participants in the active and placebo SMT arms, respectively. BIs among outcome assessors suggested adequate blinding at both study visits (active SMT: 0.08 [− 0.05 to 0.20] and 0.03 [− 0.11 to 0.16]; placebo SMT: − 0.12 [− 0.24 to 0.00] and − 0.07 [− 0.21 to 0.07]). BIs varied by participant levels of SMT lifetime experience and recent LBP. Participants and outcome assessors described different factors contributing to their beliefs.

**Conclusions:**

Adequate blinding of participants assigned to active SMT may not be feasible with the intervention protocol studied, whereas blinding of participants in the placebo SMT arm may be feasible. Blinding of outcome assessors seemed adequate. Further methodological work on blinding of SMT is needed.

**Trial registration number:**

NCT05778396.

**Supplementary Information:**

The online version contains supplementary material available at 10.1186/s12998-024-00561-0.

## Introduction

Blinding of participants and outcome assessors is essential for mitigating performance and information biases in placebo-controlled randomised controlled trials (RCTs) [1]. Yet, blinding can be challenging to achieve and maintain in RCTs of physical interventions for low back pain (LBP) [2]. After randomisation, participants and outcome assessors may come to correctly identify the intervention assigned [2–4].

Despite recent efforts to improve the reporting of blinding in placebo-controlled trials [5] and to quantify placebo effects in three-armed trials [6, 7], blinding status of participants and outcome assessors is largely unknown in RCTs of spinal manual therapy (SMT) [8]. In addition, it remains unclear if blinding may vary by levels of key baseline characteristics, and which factors may contribute to beliefs about intervention assigned. Blinding feasibility trials before larger placebo-controlled RCTs are advisable to evaluate the comparability of active and placebo interventions and identify potential threats to blinding requiring protocol changes [9, 10].

To inform the SMT blinding methods of a two-parallel-group, double-placebo controlled RCT comparing SMT versus corticosteroid nerve root injection for lumbosacral radicular pain—the SALuBRITY RCT (ISRCTN87156139 [11])—we carried out a blinding feasibility trial. Our main objectives were (1) to assess the feasibility of blinding participants randomly allocated to either active or placebo SMT intervention protocols, and (2) to assess the feasibility of blinding outcome assessors. Additional objectives were to explore (3) blinding by levels of SMT lifetime experience (i.e., received SMT in the past [yes/no]; 'SMT lifetime experience' hereafter) and recent LBP (i.e., 3 or greater average in the past four weeks on a 0 out of 10, Numeric Rating Scale [NRS] [yes/no]; ‘recent LBP’ hereafter), and (4) factors contributing to participant and outcome assessor beliefs about assigned intervention.

## Methods

### Trial design, recruitment, and participants

The trial protocol and statistical analysis plan are available [12]. This was an investigator-initiated, two-parallel-arm (allocation ratio 1:1), single-centre, placebo-controlled, blinding feasibility RCT. Our blinding feasibility trial was approved by the ethics committee of Canton Zurich (BASEC number: 2023-00381) and is reported in line with the CONSORT 2010 extension to pilot and feasibility trials [13] (see sChecklist 1 in Supplement).

We recruited participants using convenience and purposive sampling. We used word-of-mouth, snowball sampling, and mass email advertisements at the University of Zurich and Balgrist University Hospital. We enrolled adults (≥ 18 years) with or without SMT lifetime experience or recent LBP. Individuals were excluded if they self-reported: a serious spinal condition (e.g., cauda equina syndrome, progressive/widespread neurological deficit, spinal cord compression, suspicion of malignancy, infection, fracture, inflammatory spine arthritis) or serious comorbidity (i.e., a medical condition preventing them from attending the clinic site or being able to undergo a study assessment or receive SMT); a history of lumbar spine surgery; being under care or in consultation with a specialist, chiropractor, physiotherapist, or osteopath for current LBP; being a manual medicine health care provider; being pregnant or breastfeeding; being involved in pending litigation related to back pain; or, already participating in another back pain research study.

### Randomisation, allocation concealment and implementation

Participants were randomised 1:1 to active or placebo SMT intervention protocols using a computer-generated randomisation sequence. The sequence was generated by an independent statistician and stored in Research Electronic Data Capture (REDCap) [14] to ensure allocation concealment. Randomisation was stratified by SMT three-month experience (i.e., received SMT in the past three months [yes/no]; ‘SMT three-month experience’ hereafter), and recent LBP. Randomisation was blocked with randomly varying blocks of sizes two and four.

Intervention providers assigned participants to active or placebo SMT interventions at study visit 1, immediately before delivering the intervention. They performed randomisation in a private study treatment room with an electronic device and shielded the screen from the participants’ view.

### Blinding

Per protocol, participants, outcome assessors, data analysts, and investigators were blinded to intervention assignment after randomisation. To safeguard the study objectives, intervention providers and outcome assessors were kept in separate spaces at the clinic site and were trained to not discuss study aspects. In addition, REDCap user right privileges were restricted to maintain blinding of intervention assignment among all trial roles in the data management system during data collection. For data analysis, the code of the intervention variable was not broken until two complete blinded versions of the analyses and interpretations were completed [15]. By nature of the interventions, intervention providers were unblinded to the SMT intervention delivered.

The study information and consent form blinded the study objectives from participants. It stated that the aim of the study was to find out whether two SMT interventions were 'practical and acceptable' for a future study in patients with back pain (see our published protocol for details [12]). Active SMT was described as an 'active or real treatment, with an unknown exact benefit'. Placebo SMT was described as ‘other manual therapy procedure that is a comparison or control treatment that is not known to have a benefit’. We intentionally avoided ‘sham’ nomenclature in our study information form [16].

### Range of motion outcome assessment procedures

To reinforce blinding of the study objectives from participants and increase credibility, eight outcome assessors—clinicians or clinicians-in-training interacting with participants—were instructed to measure range of motion (ROM) outcomes with a phone device immediately before and after each study visit. Outcome assessors used the iOS application Measure® (iOS version 16.0.2, iPhone® model X, Apple Inc., California, United States) to capture ROM outcomes at each study visit. Per protocol, participants were blinded to these device-measured observer-reported ROM outcomes (see protocol [12]).

### Interventions

Details about the SMT interventions are available in our published protocol [12]. SMT was conceptualised as hands-on treatment directed towards the spine that included manipulation and mobilisation [17]. Nine chiropractors (mean age, 45.8 years [SD, 13.5 years]; 2 women [22.2%]; mean clinical experience, 17.4 years [SD, 10.0 years]) were trained to deliver intervention protocols according to prespecified standard operating procedures (see protocol [12]). Training involved a two-hour in-person session and additional online modules with videos of the intervention protocols and tailored REDCap tutorials.

Participants attended two study visits for their assigned intervention, on average seven days apart (mean days: 7.4 days [SD, 3.7 days]). The time between intervention sessions accommodated participant convenience and appointment availability at the clinic site. Participants’ concomitant care or information seeking was neither monitored nor restricted outside of the trial.

### Active SMT

Participants in the active SMT arm received (1) side-lying lumbar manipulation, (2) prone lumbar mobilisation, and (3) prone thoracic manipulation—all delivered with therapeutic intent.

The proposed ‘active’ element was the high-velocity, low-amplitude thrust through the target motion segments for procedures (1) and (3). For procedure (2), the proposed ‘active’ elements were the pressure, flexion and distraction components through the target motion segment.

### Placebo SMT

Careful consideration was given to the cognitive credibility of the placebo SMT intervention [18]. Participants received (1) placebo side-lying lumbar manipulation (i.e., low-velocity broad gluteal push manoeuvre), (2) placebo prone lumbar mobilisation (i.e., minimal distraction and light touch), and (3) placebo prone thoracic manipulation (i.e., push manoeuvres to the scapulae)—all performed without therapeutic intent. This placebo SMT intervention was informed by previous work [19].

### Outcomes

The primary outcome was blinding of participants, as measured by the Bang blinding index (BI) [20, 21] at two study visits. Immediately after receiving interventions, participants were asked 'Which treatment do you believe you received?', with five response options: 'Strongly believe I received the genuine treatment', 'Somewhat believe I received the genuine treatment', 'Somewhat believe I received the control treatment', 'Strongly believe I received the control treatment', and 'I do not know which treatment I received'. The Bang BI is arm-specific and chance-corrected and ranges from − 1 (all incorrect beliefs) to 1 (all correct beliefs). It can be interpreted as the proportion of correct beliefs within an intervention arm, beyond correct beliefs that would be expected by chance alone. A value of 0 is suggestive of 'perfect' blinding and is returned with an equal proportion of correct and incorrect beliefs (i.e., 'random guessing'). An arm-specific BI between − 0.3 and 0.3 was prespecified in our protocol [12] as suggestive of 'adequate' blinding [12, 22]. A sum BI was calculated measuring the between-arm difference in proportions of the same belief. For sum BI, a value of 0 is returned when an equal proportion of participants in both arms believe they received an active intervention (i.e., 'ideal' blinding).

### Secondary outcomes

The first secondary outcome was blinding of participants using an alternative BI—the James BI [23]. James BI provides a measure of study-level blinding since it combines all participant beliefs (i.e., both arms of the trial). The same belief data used for the Bang BI were used for the estimation of the James BI. The James BI measures disagreement beyond chance and returns a value between 0 and 1. A value of 1 is returned when all beliefs are 'Do not know which treatment I received' (i.e., complete blinding). A value of 0 is returned when all beliefs correctly match actual intervention assigned (i.e., complete unblinding). When 50% of beliefs are correct, and 50% incorrect, James BI returns 0.5.

Other secondary outcomes were outcome assessor blinding (Bang and James BIs), and factors contributing to beliefs about intervention assignment among all participants and outcome assessors. Participants’ credibility and expectancy of interventions were measured at the second study visit with the Credibility/Expectancy Questionnaire (CEQ) in either English [24] or German. To anchor the CEQ on clinically relevant information, participants were asked to imagine a hypothetical scenario in which they were experiencing uncomfortable pain and received their assigned study intervention for eight weeks before the CEQ assessment.

Other prespecified outcomes were included to blind the study objectives from participants. These were change in ROM [25], as well as participant-reported general health [26], satisfaction with care, back function [27, 28], and change in mid back pain and LBP intensity and function.

Intervention case report forms were completed by intervention providers in REDCap at each study visit, collecting data on: participant tolerability of intervention, intervention component fidelity, and quality of intervention delivery relative to the protocol. Adverse events were also recorded in REDCap from intervention providers and participants at each visit. Intervention providers and participants recorded adverse events in open text fields. Adverse events were documented following the Clinical Trials Ordinance applicable to the Federal Act on Research involving Human Beings (HRA, RS 810.30).

### Statistical analysis

A precision-based approach was used to consider the sample size using the Bang BI primary outcome (i.e., width of the 95% CI) [29]. We estimated that for a sample size of 26 participants per arm, the 95% CI width for the arm-specific Bang BI would be 0.45 points (see Eq. 1 in [30]). Anticipating up to 15% attrition, we aimed to recruit at least 30 participants per arm. Minimum recruitment targets for the desired precision were met, and interest in trial participation was sufficient to aim for a larger sample size with more precise estimates of the primary outcome.

Primary blinding analyses were conducted following an intention-to-treat principle with the R package BI, version 1.1.0 [31, 32]. Blinding assessment information was descriptively analysed and tabulated with numbers and percentages. To explore blinding of participants and outcome assessors by levels of SMT lifetime experience and recent LBP, blinding assessments were stratified. Within- and between-arm changes in pain intensity and ROM were described, although no statistical tests were performed.

Factors contributing to beliefs about intervention assignment were interpreted using a qualitative thematic analysis [33]. Three blinded investigators independently assessed and grouped responses by consensus.

To account for minimal deviations due to intervention providers accidentally not delivering the intervention as allocated (i.e., delivering placebo SMT instead of allocated active SMT, or delivering active SMT instead of allocated placebo SMT), we complemented our intention-to-treat blinding analysis with an as-treated analysis for the primary outcome. Two additional post hoc primary blinding analyses were performed—one by levels of participant gender and the other by individual intervention provider. A sensitivity analysis removed participants with any protocol deviation.

### Patient and public involvement and dissemination

Patients and members of the public were not involved in this blinding feasibility trial due to resource constraints. Yet, patient and clinician perspectives from other preliminary work [34] are helping to inform the SALuBRITY trial protocol and indirectly informed this methodological trial. Also, patient representatives were involved in developing the CoPPS Statement [5] and found blinding and placebo-controlled trials acceptable.

## Results

**Fig. 1:**
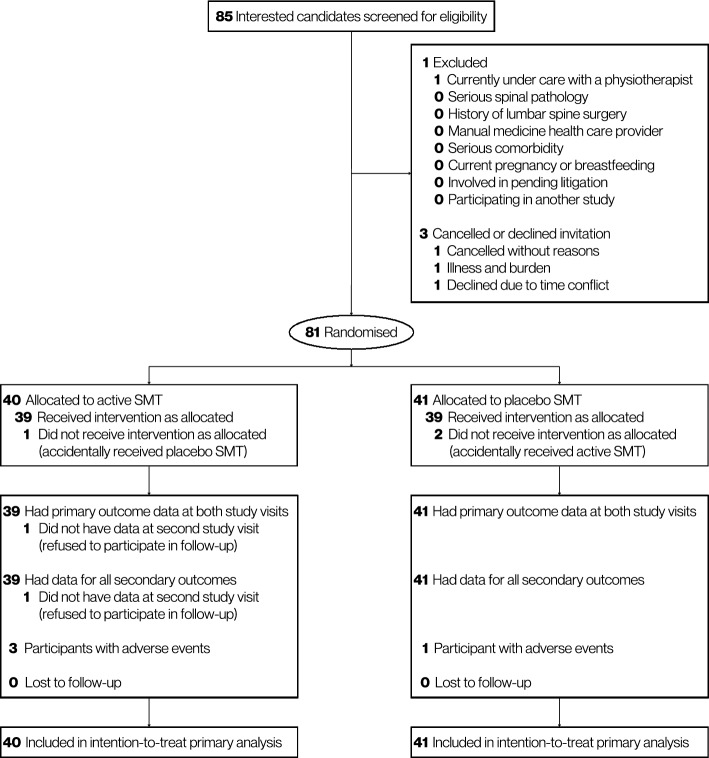
Participant recruitment, randomisation, and follow-up

Between 4 and 25 April 2023, 85 people were screened for eligibility and 81 were randomised. Of these, 40 participants were randomly allocated to active SMT and 41 to placebo SMT (Fig. 1). One participant in the active SMT arm refused to participate in follow-up visit and withdrew from the study after study visit 1. At study visit 2, there were complete data for 80 participants (99%).

Baseline characteristics were similar between participants in the two intervention arms (Table 1), except gender (active SMT, 15 female [38%]; placebo SMT, 27 [66%]). The study population had a mean age of 39 years (SD, 13 years) and a mean body mass index of 24.2 (SD, 3.8). At baseline, 41 participants (51%) reported SMT lifetime experience, and 29 (36%) recent LBP (Table 1, sTable 1).

**Table 1:** Participant characteristics at baseline

Characteristic	Overall (*n* = 81)	Active SMT (*n* = 40)	Placebo SMT (*n* = 41)
*Gender, no. (%)*			
Women	42 (52)	15 (38)	27 (66)
Men	39 (48)	25 (62)	14 (34)
Other or prefer not to say	0 (0)	0 (0)	0 (0)
Age—yrs, mean (SD)	39.1 (13.2)	39.3 (13.0)	39.0 (13.6)
Weight—kg, mean (SD)	72.3 (14.3)	72.9 (13.1)	71.7 (15.6)
Height—cm, mean (SD)	172.4 (9.7)	174.3 (9.9)	170.5 (9.4)
Body mass index—kg/m^2^, mean (SD)	24.2 (3.8)	23.8 (2.6)	24.5 (4.7)
SMT lifetime experience, No. (%)^a^	41 (51)	19 (48)	22 (54)
Recent LBP, no. (%)^b^	29 (36)	12 (30)	17 (41)
SMT three-month experience, No. (%)^c^	4 (5)	2 (5)	2 (5)
LBP average intensity in past four weeks—NRS, mean (SD)^d^	2.4 (2.5)	2.0 (2.2)	2.7 (2.7)
*ROM—degrees, mean (SD)*^e^			
Flexion	116.8 (17.4)	114.7 (19.2)	118.8 (15.6)
Extension	32.3 (11.9)	32.2 (11.2)	32.3 (12.7)
Total	149.0 (24.2)	147.0 (23.1)	151.1 (25.3)

*cm* centimeters, *kg* kilograms, *LBP* low back pain, *No.* number, *NRS* Numeric Rating Scale, *ROM* range of motion; *SD*, standard deviation; *SMT*, spinal manual therapy; *yrs,* years

^a^Received SMT in the past

^b^3 or greater average in the past four weeks on a 0 (no pain) out of 10 (worst pain imaginable), NRS

^c^Received SMT in the past three months

^d^ Ranges from 0 (no pain) to 10 (worst pain imaginable); for the NRS, a score of 3 or less indicates mild pain; a score of 4 to 6, moderate pain; and a score of 7 or greater, severe pain

^e^ROM measurements using the iOS application Measure® (iOS version 16.0.2, iPhone® model X, Apple Inc., California, United States) [25]

### Blinding of participants

**Fig. 2:**
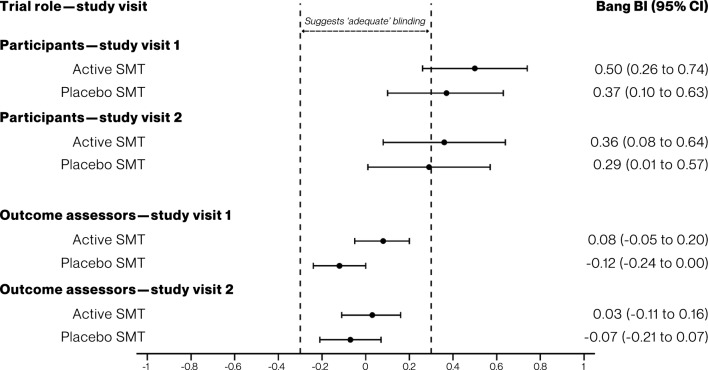
Blinding of participants and outcome assessors

At study visit 1, 50% of participants in the active SMT arm (Bang BI: 0.50 [95% confidence interval (CI), 0.26 to 0.74]) and 37% in the placebo SMT arm (0.37 [95% CI, 0.10 to 0.63]) had a correct belief about their assigned intervention, beyond chance. At study visit 2, Bang BIs were 0.36 (0.08 to 0.64) and 0.29 (0.01 to 0.57) for participants in the active and placebo arms, respectively (Table 2**, **Fig. 2, sTable 2). James BIs for study participants were 0.35 (0.24 to 0.45) and 0.37 (0.27 to 0.48) for study visits 1 and 2. After accounting for three protocol deviations (active SMT, 1 [2.5%]; placebo-control SMT, 2 [4.9%]) due to intervention providers accidentally not delivering the intervention as allocated, the as-treated analysis was consistent with the intention-to-treat primary analysis (sTable 3).

**Table 2:** Primary and secondary outcomes

**Outcome**	Active SMT (*n* = 40)	Placebo SMT (*n* = 41)	Overall (95% CI)
*Primary outcome*			
Bang BI of participants—study visit 1^a^	0.50 (0.26 to 0.74)	0.37 (0.10 to 0.63)	Sum BI, 0.87 (0.51 to 1.23)
Bang BI of participants—study visit 2^a^	0.36 (0.08 to 0.64)	0.29 (0.01 to 0.57)	Sum BI, 0.65 (0.26 to 1.04)
*Secondary outcomes*			
James BI of participants—study visit 1^b^			0.35 (0.24 to 0.45)
James BI of participants—study visit 2^b^			0.37 (0.27 to 0.48)
Bang BI of outcome assessors—study visit 1^c^	0.08 (− 0.05 to 0.20)	− 0.12 (− 0.24 to − 0.00)	Sum BI, − 0.04 (− 0.22 to 0.14)
Bang BI of outcome assessors—study visit 2^c^	0.03 (− 0.11 to 0.16)	− 0.07 (− 0.21 to 0.07)	Sum BI, − 0.04 (− 0.23 to 0.15)
James BI of outcome assessors—study visit 1^c^			0.93 (0.87 to 0.98)
James BI of outcome assessors —study visit 2^c^			0.91 (0.85 to 0.97)

Other participant outcomes	Active SMT (n = 40)	Placebo SMT (n = 41)	Effect (95% CI)
Credibility—study visit 2, mean (SD)^d^	6.40 (1.76)	4.91 (2.50)	MD, 1.49 (0.55 to 2.43)
Expectancy—study visit 2, mean (SD)^e^	6.17 (1.65)	4.84 (2.54)	MD, 1.33 (0.40 to 2.26)
Credibility/Expectancy—study visit 2, mean (SD)^f^	6.29 (1.66)	4.87 (2.49)	MD, 1.42 (0.50 to 2.34)
*Change in ROM—degrees, mean (SD)*^g^			
Flexion	− 2.72 (11.65)	0.07 (10.36)	MD, − 2.79 (− 7.63 to 2.05)
Extension	− 0.03 (6.39)	0.73 (8.57)	MD, − 0.76 (− 4.06 to 2.54)
Total	− 2.74 (13.98)	0.80 (14.99)	MD, − 3.54 (− 9.89 to 2.81)

*BI* blinding index, *CEQ* Credibility/Expectancy Questionnaire, *MD* mean difference

^a^From responses to the questions 'Which treatment do you believe you received?'; Bang BI ranges from –1 (all incorrect beliefs) to 1 (all correct beliefs); values between − 0.3 and 0.3 may be suggestive of 'adequate' blinding

^b^From responses to the question 'Which treatment do you believe you received?'; James BI ranges from 0 (all correct beliefs) to 1 (all 'Do not know' beliefs); when 50% of beliefs are correct, and 50% incorrect, James BI returns 0.5

^c^From responses to the question 'Which treatment do you believe this participant received?'

^d^Credibility subcomponent of the CEQ (items 1 to 3) [24]; ranges from 1 (least credible) to 9 (most credible)

^e^Expectancy subcomponent of the CEQ (items 4 to 6) [24]; ranges from 1 (lowest expectancy) to 9 (highest expectancy)

^f^Credibility and expectancy subcomponents of the CEQ, combined [24] (items 1 to 6); ranges from 1 (least credible and lowest expectancy) to 9 (most credible and highest expectancy)

^g^Change in device-measured observer-reported ROM measurements between baseline and post-study visit 2 using the iOS application Measure® (iOS version 16.0.2, iPhone® model X, Apple Inc., California, United States) [24]; higher positive values indicate larger positive change in ROM

### Credibility and expectancy

Mean CEQ scores at the second study visit were 6.29 (SD, 1.66) for the active SMT arm and 4.87 (SD, 2.49) for the placebo SMT arm. Mean credibility scores were 6.40 (SD, 1.76) for the active and 4.91 (SD, 2.50) for the placebo SMT arm, whereas corresponding expectancy scores were 6.17 (SD, 1.65) and 4.84 (SD, 2.54) (Table 2).

### Blinding of outcome assessors

Outcome assessors at study visit 1 had Bang BIs of 0.08 (95% CI, − 0.05 to 0.20) and − 0.12 (95% CI, − 0.24 to − 0.00) for their beliefs about participant intervention assignment in the active and placebo SMT arms, respectively. At study visit 2, Bang BIs for outcome assessors were 0.03 (95% CI, − 0.11 to 0.16) and − 0.07 (95% CI, − 0.21 to 0.07). James BIs for outcome assessors were 0.93 (0.87 to 0.98) and 0.91 (0.85 to 0.97) at study visits 1 and 2, respectively (Table 2, Fig. 2, sTable 4). Blinding estimates for outcome assessors were similar in the as-treated analysis (sTable 5).

### Exploratory analyses

#### Blinding by SMT lifetime experience

Among participants with SMT lifetime experience, Bang BIs were 0.53 (0.19 to 0.86) in the active and 0.18 (− 0.21 to 0.57) in the placebo SMT arm at study visit 1; 0.47 (0.11 to 0.84) and 0.14 (− 0.27 to 0.54) at study visit 2 (sTable 6).

Among participants without SMT lifetime experience, Bang BIs were 0.48 (0.14 to 0.82) and 0.58 (0.24 to 0.92) in the active and placebo SMT arms at study visit 1; 0.25 (− 0.16 to 0.66) and 0.47 (0.11 to 0.84) at study visit 2 (sTable 7).

Blinding estimates for outcome assessors suggested adequate blinding after stratifying by participant SMT lifetime experience (sTable 8).

#### Blinding by recent LBP

Among participants with recent LBP, Bang BIs were 0.33 (− 0.15 to 0.81) and 0.47 (0.07 to 0.87) within the active and placebo SMT arms at study visit 1; 0.50 (0.07 to 0.93) and 0.59 (0.22 to 0.95) at study visit 2 (sTable 9).

Among participants without recent LBP, Bang BIs were 0.57 (0.30 to 0.84) and 0.29 (− 0.06 to 0.65) in the active and placebo SMT arms at study visit 1; 0.30 (− 0.06 to 0.65) and 0.08 (− 0.30 to 0.46) at study visit 2 (sTable 10).

After stratifying by participant LBP experience, blinding of outcome assessors remained adequate (sTable 11).

#### Factors contributing to beliefs about assigned intervention

Participants and outcome assessors reported different factors contributing to their beliefs about the assigned intervention. At study visit 1, participants commented on general effects following the intervention, intervention elements, and their experience with SMT. At study visit 2, participants described general effects following the intervention, intervention elements, and shared their perspectives about the comparability of the two study visits (Table 3**,** sCodebook 1).

**Table 3:** Factors contributing to beliefs about the assigned intervention

*Trial role*	Study visit 1, no (%)		Study visit 2, no. (%)
Theme		Theme	
*Participants*			
General effects	24 (25)	General effects	30 (24)
Intervention	14 (14)	Intervention	29 (23)
Experience	13 (13)	Comparison of intervention sessions	27 (22)
Musculoskeletal effect	11 (11)	Manual element	15 (12)
Movement	7 (7)	Movement	7 (6)
Sound	7 (7)	Experience	6 (5)
Uncertainty	7 (7)	Impression	6 (5)
Manual element	6 (6)	Musculoskeletal effect	5 (4)
Pain or discomfort	4 (4)	Sound	5 (4)
Impression	3 (3)	Uncertainty	4 (3)
Miscellaneous	2 (2)	Pain or discomfort	3 (2)
–	–	Expectations	1 (1)
–	–	External factor	1 (1)
*Outcome assessors*			
No change (unspecific)	56 (64)	No change (unspecific)	27 (41)
Uncertainty	15 (17)	Uncertainty	15 (23)
Movement	7 (8)	Movement	11 (17)
Cues	6 (7)	Participant expression	7 (11)
Participant expression	3 (3)	Cues	6 (10)

Outcome assessors reported 'no change' in participants as a predominant factor contributing to their beliefs about the assigned intervention. Although outcome assessors considered the ROM of participants they were measuring, they expressed uncertainty at both study visits (Table 3, sCodebook 2).

#### Other outcomes

Other participant outcomes were comparable between intervention arms (sTable 12).

Intervention provider outcomes were also similar between intervention arms. In both SMT arms, intervention providers reported high participant tolerability, intervention component fidelity, and quality of intervention delivery (sTable 13).

#### Adverse events

Six adverse events (active SMT: 4; placebo SMT: 2) were reported from three participants (7.5%) in the active SMT arm and one participant (2.4%) in the placebo SMT arm—all deemed mild and possibly related to interventions. Of the six adverse events, four (66.7%) were musculoskeletal (i.e., increased back pain or discomfort) and two (33.3%) were vestibular (i.e., dizziness) (sTable 14). There were no serious adverse events.

#### Post hoc analyses

Blinding estimates varied by participant gender and intervention provider (sTable 15, sTable 16). The sensitivity analysis excluded nine participants with protocol deviations (active SMT, 5 [12.5%]; placebo SMT, 4 [9.8%]) and yielded results consistent with the primary analysis (sTable 17).

## Discussion

In this blinding feasibility randomised trial comparing two study visits for either active or placebo SMT interventions, on average seven days apart, adequate blinding of participants allocated to active SMT was not observed. Blinding of participants allocated to placebo SMT may be adequate at the second study visit. Blinding of outcome assessors was feasible and promising. Exploratory analyses suggested that participants with SMT lifetime experience were not better at identifying their intervention assigned. Participants without recent LBP achieved adequate blinding at the second study visit. Different factors contributed to participant and outcome assessor beliefs about the assigned intervention.

Our findings are informative and have implications for placebo-controlled RCTs of SMT, such as the SALuBRITY trial [11]. First, SMT intervention protocols warrant testing and blinding assessment to improve blinding feasibility among participants, and balance credibility and expectancy across intervention arms. Second, our BI estimates for outcome assessors suggest that some trial roles may achieve and maintain adequate blinding after randomisation. Third, participants with SMT lifetime experience in our study were not better at identifying their assigned intervention. This suggests, counterintuitively, that intervention experience may not inherently compromise blinding. Fourth, blinding of participants improved at the second study visit, which suggests that participants may not necessarily develop more accurate beliefs seven days after randomisation and after two study visits for the intervention assigned. Fifth, the BIs most suggestive of adequate blinding at the second study visit were observed among participants without recent LBP, which is of methodological interest but of limited clinical relevance for the design of RCTs of SMT. Factors contributing to intervention beliefs among participants provide clues about the influence of general effects and key intervention aspects on blinding feasibility. A prominent factor emerging at the second study visit was 'comparison of intervention sessions', which may motivate trialists to carefully consider the ideal number and timing of blinding assessments. Altogether, these factors have potential to improve the design, quality, and fidelity of future physical RCT intervention protocols.

Our blinding feasibility findings among participants can be understood in light of a recent meta-analysis of placebo-controlled RCTs for back pain [3, 35]. Compared to the pooled blinding estimates for physical interventions within this meta-analytical work and a recent placebo-controlled RCT of SMT [36], blinding of participants seemed less adequate in our study. Clinical heterogeneity (i.e., related to specific interventions or patient characteristics) and methodological heterogeneity (i.e., related to study design and risk of bias) may account for variability of blinding estimates across RCTs of SMT [37–39]. Our credibility and expectancy findings are in line with those of a recent RCT of SMT for back pain [40].

Our study has strengths. First, we prespecified standard operating procedures and training for outcome assessors and intervention providers to increase protocol adherence, maximise intervention fidelity, and attempt to balance contextual effects. Second, we fostered impartiality by blinding the trial objectives from participants and outcome assessors. In addition, we completed two blinded versions of the analyses before breaking the intervention code. Third, we used a manual placebo SMT intervention as comparator and prespecified in our protocol the 'active' element it lacked. Fourth, our trial was carried out in a real-world community-based clinic setting and had high retention of participants for the two study visits. Fifth, we included outcome assessors in our blinding assessment, gaining a more comprehensive understanding of blinding. Sixth, we assessed blinding at two study visits, on average seven days apart, allowing us to investigate changes in beliefs about the assigned intervention. Seventh, we included a qualitative thematic analysis to explore factors that may influence beliefs about the assigned intervention.

Our study also has limitations. First, we deviated from the original protocol and adapted the operational definition of SMT experience for exploratory stratified analyses, from SMT three-month experience to SMT lifetime experience. Second, despite training of intervention providers, three participants accidentally did not receive the intervention as allocated. We complemented our intention-to-treat blinding analysis with sensitivity analyses to explore the impact of these allocation deviations. Third, despite proper implementation of randomisation procedures, we observed an imbalance in participant gender at baseline. We performed a post-hoc blinding analysis by levels of participant gender to explore potential variability of blinding estimates in men and women. Fourth, our study population had, on average, mild intensity LBP at baseline, which may limit the generalisability and transportability of our blinding estimates.

## Conclusion

Adequate blinding of participants assigned to active SMT may not be feasible with the intervention protocol studied, whereas blinding of participants in the placebo SMT arm may be feasible. Blinding of outcome assessors seemed feasible and adequate. Despite inherent challenges, further methodological work on blinding of SMT interventions is needed.

## Supplementary Information

Additional file 1.

## Data Availability

The collected data files and other materials, including full protocol, study forms, dataset, and statistical code, are available on reasonable request from the corresponding or first author.

## Abbreviations

BASEC: Business Administration System for Ethics Committees
CI: Confidence interval
HRA, RS: Human Research Act, Systematic Compilation of Federal Legislation
RCT: Randomised controlled trial
REDCap: Research electronic data capture
ROM: Range of motion
SALuBRITY: Spinal manual therapy versus nerve root injection for lumbosacral radicular pain
SD: Standard deviation
SMT: Spinal manual therapy
