# Supplementary material for: 'Which treatment do you believe you received?' A randomised blinding feasibility trial of spinal manual therapy
Source: Chiropr Man Therap. 2025 Jan 14;33:4. doi: 10.1186/s12998-024-00561-0 (PMC11730787; doi:10.1186/s12998-024-00561-0)
Supplement: Supplementary file 1 — Additional file 1. [file 12998_2024_561_MOESM1_ESM.pdf]

Supplement

Muñoz Laguna J, Kurmann A, Hofstetter L, Nyantakyi E, Braun J, Clack L, et al. ‘Which treatment do you believe you received?’ A randomised blinding feasibility trial of spinal manual therapy. Chiropr Man Ther. 2024

Contents

Section 1. CONSORT 2010 ... 2
- sChecklist 1. CONSORT 2010 extension to randomised pilot and feasibility trials ... 2

Section 2. Additional participant characteristics ... 5
- sTable 1. Additional participant characteristics at baseline ... 5

Section 3. Blinding assessment tables ... 6
- sTable 2. Blinding assessment of participants (intention-to-treat, ITT) ... 6
- sTable 3. Blinding assessment of participants (as-treated, AT) ... 6
- sTable 4. Blinding assessment of outcome assessors (ITT) ... 7
- sTable 5. Blinding assessment of outcome assessors (AT) ... 7
- sTable 6. Blinding assessment of participants with SMT lifetime experience (ITT) ... 8
- sTable 7. Blinding assessment of participants without SMT experience (ITT) ... 8
- sTable 8. Blinding assessment of outcome assessors in participants with SMT experience (ITT) ... 9
- sTable 9. Blinding assessment of participants with recent LBP (ITT) ... 10
- sTable 10. Blinding assessment of participants without recent LBP (ITT) ... 10
- sTable 11. Blinding assessment of outcome assessors by levels of participant LBP (ITT) ... 11

Section 4. Codebooks for qualitative thematic analysis ... 12
- sCodebook 1. Categories, sub-categories, definitions and coded examples (participants) ... 12
- sCodebook 2. Categories, sub-categories, definitions and coded examples (outcome assessors) ... 18

Section 5. Additional supplemental tables ... 19
- sTable 12. Other participant outcomes ... 19
- sTable 13. Clinician-reported intervention delivery outcomes ... 20
- sTable 14. Adverse events ... 20

Section 6. Blinding assessment tables for post hoc analyses ... 21
- sTable 15. Blinding assessment by levels of gender (ITT) ... 21
- sTable 16. Blinding assessment by levels of intervention provider (ITT) ... 22
- sTable 17. Blinding assessment of participants without any protocol deviation (sensitivity) ... 26

Section 1. CONSORT 2010

sChecklist 1. CONSORT 2010 extension to randomised pilot and feasibility trials

Section/Topic	Item No	Checklist item	Reported
**Title and abstract**			
	1a	Identification as a pilot or feasibility randomised trial in the title	✓
	1b	Structured summary of pilot trial design, methods, results, and conclusions (for specific guidance see CONSORT abstract extension for pilot trials)	✓
**Introduction**			
Background and objectives	2a	Scientific background and explanation of rationale for future definitive trial, and reasons for randomised pilot trial	✓
	2b	Specific objectives or research questions for pilot trial	✓
**Methods**			
Trial design	3a	Description of pilot trial design (such as parallel, factorial) including allocation ratio	✓
	3b	Important changes to methods after pilot trial commencement (such as eligibility criteria), with reasons	✓
Participants	4a	Eligibility criteria for participants	✓
	4b	Settings and locations where the data were collected	✓
	4c	How participants were identified and consented	✓
Interventions	5	The interventions for each group with sufficient details to allow replication, including how and when they were actually administered	✓
Outcomes	6a	Completely defined prespecified assessments or measurements to address each pilot trial objective specified in 2b, including how and when they were assessed	✓
	6b	Any changes to pilot trial assessments or measurements after the pilot trial commenced, with reasons	✓
	6c	If applicable, prespecified criteria used to judge whether, or how, to proceed with future definitive trial	✓
Sample size	7a	Rationale for numbers in the pilot trial	✓

	7b	When applicable, explanation of any interim analyses and stopping guidelines	NA
Randomisation:			
Sequence generation	8a	Method used to generate the random allocation sequence	✓
	8b	Type of randomisation(s); details of any restriction (such as blocking and block size)	✓
Allocation concealment mechanism	9	Mechanism used to implement the random allocation sequence (such as sequentially numbered containers), describing any steps taken to conceal the sequence until interventions were assigned	✓
Implementation	10	Who generated the random allocation sequence, who enrolled participants, and who assigned participants to interventions	✓
Blinding	11a	If done, who was blinded after assignment to interventions (for example, participants, care providers, those assessing outcomes) and how	✓
	11b	If relevant, description of the similarity of interventions	✓
Statistical methods	12	Methods used to address each pilot trial objective whether qualitative or quantitative	✓
**Results**			
Participant flow (a diagram is strongly recommended)	13a	For each group, the numbers of participants who were approached and/or assessed for eligibility, randomly assigned, received intended treatment, and were assessed for each objective	✓
	13b	For each group, losses and exclusions after randomisation, together with reasons	✓
Recruitment	14a	Dates defining the periods of recruitment and follow-up	✓
	14b	Why the pilot trial ended or was stopped	NA
Baseline data	15	A table showing baseline demographic and clinical characteristics for each group	✓
Numbers analysed	16	For each objective, number of participants (denominator) included in each analysis. If relevant, these numbers should be by randomised group	✓
Outcomes and estimation	17	For each objective, results including expressions of uncertainty (such as 95% confidence interval) for any estimates. If relevant, these results should be by randomised group	✓
Ancillary analyses	18	Results of any other analyses performed that could be used to inform the future definitive trial	✓
Harms	19	All important harms or unintended effects in each group (for specific guidance see CONSORT for harms)	✓
	19a	If relevant, other important unintended consequences	NA
**Discussion**			

Limitations	20	Pilot trial limitations, addressing sources of potential bias and remaining uncertainty about feasibility	✓
Generalisability	21	Generalisability (applicability) of pilot trial methods and findings to future definitive trial and other studies	✓
Interpretation	22	Interpretation consistent with pilot trial objectives and findings, balancing potential benefits and harms, and<br>considering other relevant evidence	✓
	22a	Implications for progression from pilot to future definitive trial, including any proposed amendments	NA
**Other information**			
Registration	23	Registration number for pilot trial and name of trial registry	✓
Protocol	24	Where the pilot trial protocol can be accessed, if available	✓
Funding	25	Sources of funding and other support (such as supply of drugs), role of funders	✓
	26	Ethical approval or approval by research review committee, confirmed with reference number	✓

Abbreviations: *NA*, not applicable

Citation: Eldridge SM, Chan CL, Campbell MJ, Bond CM, Hopewell S, Thabane L, et al. CONSORT 2010 statement: extension to randomised pilot and feasibility trials. BMJ. 2016;355

Section 2. Additional participant characteristics

sTable 1. Additional participant characteristics at baseline

Characteristic	Overall (n = 81)	Active SMT (n = 40)	Placebo SMT (n = 41)
Overweight or obese, No. (%)	26 (33)	15 (38)	11 (28)
LBP intensity in past week—NRS, mean (SD)	2.4 (2.6)	2.1 (2.4)	2.6 (2.8)
Current LBP intensity—NRS, mean (SD)	1.7 (2.2)	1.6 (2.1)	1.9 (2.4)
MBP intensity in past week—NRS, mean (SD)	1.6 (2.3)	1.7 (2.4)	1.4 (2.2)
Current MBP intensity—NRS, mean (SD)	1.3 (2.1)	1.1 (1.8)	1.4 (2.4)
Back function—NRS, mean (SD)	7.2 (2.0)	7.1 (2.0)	7.3 (2.0)
Ache, pain, discomfort in low back, No. (%)			
None	35 (43)	19 (48)	16 (39)
Yes—Slightly	21 (26)	10 (25)	11 (27)
Yes—Moderately	18 (22)	8 (20)	10 (24)
Yes—Very	7 (9)	3 (8)	4 (10)
Ache, pain, discomfort in mid back, No. (%)			
None	52 (64)	26 (65)	26 (63)
Yes—Slightly	12 (15)	6 (15)	6 (15)
Yes—Moderately	14 (17)	7 (18)	7 (17)
Yes—Very	3 (4)	1 (2)	2 (5)
Self-rated flexibility, No. (%)			
Very poor/Poor	12 (15)	6 (15)	6 (15)
Average	25 (31)	13 (32)	12 (29)
Good	30 (37)	16 (40)	14 (34)
Very good	14 (17)	5 (13)	9 (22)
Self-rated general health, No. (%)			
Fair/Poor	0 (0)	0 (0)	0 (0)
Good	16 (20)	7 (18)	9 (22)
Very good	49 (60)	24 (60)	25 (61)
Excellent	16 (20)	9 (22)	7 (17)

Abbreviations: *LBP*, low back pain; *MBP*, mid back pain; *No.*, number; *NRS*, Numeric Rating Scale; *SD*, standard deviation

Section 3. Blinding assessment tables

sTable 2. Blinding assessment of participants (intention-to-treat, ITT)

	Beliefs						
Assignment	Strongly believe genuine	Somewhat believe genuine	Somewhat believe control	Strongly believe control	Do not know	Total	Bang BI
Study visit 1							
Active SMT	4 (10.0%)	23 (57.5%)	6 (15.0%)	1 (2.5%)	6 (15.0%)	40	0.50 (0.26 to 0.74)
Placebo SMT	2 (4.9%)	9 (22.0%)	16 (39.0%)	10 (24.4%)	4 (9.8%)	41	0.37 (0.10 to 0.63)
Total	6	32	22	11	10	81	
Study visit 2							
Active SMT	8 (20.5%)	17 (43.6%)	9 (23.1%)	2 (5.1%)	3 (7.7%)	39	0.36 (0.08 to 0.64)
Placebo SMT	0	13 (31.7%)	15 (36.6%)	10 (24.4%)	3 (7.3%)	41	0.29 (0.01 to 0.57)
Total	8	30	24	12	6	80	

sTable 3. Blinding assessment of participants (as-treated, AT)

	Beliefs						
Assignment	Strongly believe genuine	Somewhat believe genuine	Somewhat believe control	Strongly believe control	Do not know	Total	Bang BI
Study visit 1							
Active SMT	4 (9.5%)	24 (57.1%)	6 (14.3%)	2 (4.8%)	6 (14.3%)	42	0.47 (0.24 to 0.72)
Placebo SMT	2 (5.1%)	8 (20.5%)	16 (41.0%)	9 (23.1%)	4 (10.3%)	39	0.38 (0.11 to 0.66)
Total	6	32	22	11	10	81	
Study visit 2							
Active SMT	8 (21.1%)	17 (44.7%)	8 (21.1%)	2 (5.3%)	3 (7.9%)	38	0.39 (0.12 to 0.67)
Placebo SMT	0	13 (31.0%)	16 (38.1%)	10 (23.8%)	3 (7.1%)	42	0.31 (0.03 to 0.59)
Total	8	30	24	12	6	80	

sTable 4. Blinding assessment of outcome assessors (ITT)

	Beliefs						
Assignment	Strongly believe genuine	Somewhat believe genuine	Somewhat believe control	Strongly believe control	Do not know	Total	Bang BI
Study visit 1							
Active SMT	0	5 (12.5%)	2 (5.0%)	0	33 (82.5.%)	40	0.08 (-0.05 to 0.20)
Placebo SMT	3 (7.3%)	3 (7.3%)	1 (2.4%)	0	34 (82.9%)	41	-0.12 (-0.24 to -0.00)
Total	3	8	3	0	67	81	
Study visit 2							
Active SMT	0	4 (10.3%)	3 (7.7%)	0	32 (82.1%)	39	0.03 (-0.11 to 0.16)
Placebo SMT	0	6 (14.6%)	3 (7.3%)	0	32 (78.0%)	41	-0.07 (-0.21 to 0.07)
Total	0	10	6	0	64	80	

sTable 5. Blinding assessment of outcome assessors (AT)

	Beliefs						
Assignment	Strongly believe genuine	Somewhat believe genuine	Somewhat believe control	Strongly believe control	Do not know	Total	Bang BI
Study visit 1							
Active SMT	0	5 (11.9%)	3 (7.1%)	0	34 (81%)	42	0.05 (-0.08 to 0.18)
Placebo SMT	3 (7.7%)	3 (7.7%)	0	0	33 (84.6%)	39	-0.15 (-0.27 to -0.04)
Total	3	8	3	0	67	81	
Study visit 2							
Active SMT	0	4 (10.5%)	3 (7.9%)	0	31 (81.6%)	38	0.03 (-0.11 to 0.16)
Placebo SMT	0	6 (14.3%)	3 (7.1%)	0	33 (78.6%)	42	-0.07 (-0.21 to 0.07)
Total	0	10	6	0	64	80	

sTable 6. Blinding assessment of participants with SMT lifetime experience (ITT)

	Beliefs						
Assignment	Strongly believe genuine	Somewhat believe genuine	Somewhat believe control	Strongly believe control	Do not know	Total	Bang BI
Study visit 1							
Active SMT	1 (5.3%)	12 (63.2%)	3 (15.8%)	0	3 (15.8%)	19	0.53 (0.19 to 0.86)
Placebo SMT	1 (4.5%)	7 (31.2%)	8 (36.4%)	4 (18.2%)	2 (9.1%)	22	0.18 (-0.21 to 0.57)
Total	2	19	11	4	5	41	
Study visit 2							
Active SMT	4 (21.1%)	9 (47.4%)	4 (21.1%)	0	2 (10.5%)	19	0.47 (0.11 to 0.84)
Placebo SMT	0	9 (40.9%)	7 (31.8%)	5 (22.7%)	1 (4.5%)	22	0.14 (-0.27 to 0.54)
Total	4	18	11	5	3	41	

sTable 7. Blinding assessment of participants without SMT experience (ITT)

	Beliefs						
Assignment	Strongly believe genuine	Somewhat believe genuine	Somewhat believe control	Strongly believe control	Do not know	Total	Bang BI
Study visit 1							
Active SMT	3 (14.3%)	11 (52.4%)	3 (14.3%)	1 (4.8%)	3 (14.3%)	21	0.48 (0.14 to 0.82)
Placebo SMT	1 (5.3%)	2 (10.5%)	8 (42.1%)	6 (31.6%)	2 (10.5%)	19	0.58 (0.24 to 0.92)
Total	4	13	11	7	5	40	
Study visit 2							
Active SMT	4	8	5	2	1	20	0.25 (-0.16 to 0.66)
Placebo SMT	0	4	8	5	2	19	0.47 (0.11 to 0.84)
Total	4	12	13	7	3	39	

sTable 8. Blinding assessment of outcome assessors in participants with SMT experience (ITT)

	Beliefs						
Assignment	Strongly believe genuine	Somewhat believe genuine	Somewhat believe control	Strongly believe control	Do not know	Total	Bang BI
Participants with SMT experience							
Study visit 1							
Active SMT	0	3 (15.8%)	1 (5.3%)	0	15 (78.9%)	19	0.11 (-0.10 to 0.31)
Placebo SMT	2 (9.1%)	2 (9.1%)	1 (4.5%)	0	17 (77.2%)	22	-0.14 (-0.33 to 0.05)
Total	2	5	2	0	32	41	
Study visit 2							
Active SMT	0	2 (10.5%)	0	0	17 (89.5%)	19	0.11 (-0.03 to 0.24)
Placebo SMT	0	3 (13.6%)	2 (9.1%)	0	17 (77.3%)	22	-0.05 (-0.24 to 0.15)
Total	0	5	2	0	34	41	
Participants without SMT experience							
Study visit 1							
Active SMT	0	2 (9.5%)	1 (4.8%)	0	18 (85.7%)	21	0.05 (-0.11 to 0.21)
Placebo SMT	1 (5.3%)	1 (5.3%)	0	0	17 (89.5%)	19	-0.11 (-0.24 to 0.03)
Total	1	3	1	0	35	40	
Study visit 2							
Active SMT	0	2 (10.0%)	3 (15.0%)	0	15 (75.0%)	20	-0.05 (-0.27 to 0.17)
Placebo SMT	0	3 (15.8%)	1 (5.3%)	0	15 (78.9%)	19	-0.11 (-0.31 to 0.10)
Total	0	5	4	0	30	39	

sTable 9. Blinding assessment of participants with recent LBP (ITT)

	Beliefs						
Assignment	Strongly believe genuine	Somewhat believe genuine	Somewhat believe control	Strongly believe control	Do not know	Total	Bang BI
Study visit 1							
Active SMT	0	7 (58.3%)	3 (25.0%)	0	2 (16.7%)	12	0.33 (-0.15 to 0.81)
Placebo SMT	1 (5.9%)	3 (17.6%)	6 (35.3%)	6 (35.3%)	1 (5.9%)	17	0.47 (0.07 to 0.87)
Total	1	10	9	6	3	29	
Study visit 2							
Active SMT	2 (16.7%)	6 (50.0%)	2 (16.7%)	0	2 (16.7%)	12	0.50 (0.07 to 0.93)
Placebo SMT	0	3 (17.6%)	7 (41.2%)	6 (35.3%)	1 (5.9%)	17	0.59 (0.22 to 0.95)
Total	2	9	9	6	3	29	

sTable 10. Blinding assessment of participants without recent LBP (ITT)

	Beliefs						
Assignment	Strongly believe genuine	Somewhat believe genuine	Somewhat believe control	Strongly believe control	Do not know	Total	Bang BI
Study visit 1							
Active SMT	4 (14.3%)	16 (57.1%)	3 (10.7%)	1 (3.6%)	4 (14.3%)	28	0.57 (0.30 to 0.84)
Placebo SMT	1 (4.2%)	6 (25.0%)	10 (41.7%)	4 (16.7%)	3 (12.5%)	24	0.29 (-0.06 to 0.65)
Total	5	22	13	5	7	52	
Study visit 2							
Active SMT	6 (22.2%)	11 (40.7%)	7 (25.9%)	2 (7.4%)	1 (3.7%)	27	0.30 (-0.06 to 0.65)
Placebo SMT	0	10 (41.7%)	8 (33.3%)	4 (16.7%)	2 (8.3%)	24	0.08 (-0.30 to 0.46)
Total	6	21	15	6	3	51	

sTable 11. Blinding assessment of outcome assessors by levels of participant LBP (ITT)

	Beliefs						
Assignment	Strongly believe genuine	Somewhat believe genuine	Somewhat believe control	Strongly believe control	Do not know	Total	Bang BI
Participants with recent LBP							
Study visit 1							
Active SMT	0	1 (8.3%)	1 (8.3%)	0	10 (83.3%)	12	0 (-0.23 to 0.23)
Placebo SMT	2 (11.8%)	1 (5.9%)	1 (5.9%)	0	13(76.5%)	17	-0.12 (-0.34 to 0.11)
Total	2	2	2	0	23	29	
Study visit 2							
Active SMT	0	1 (8.3%)	1 (8.3%)	0	10 (83.3%)	21	0 (-0.23 to 0.23)
Placebo SMT	0	1 (5.9%)	3 (17.6%)	0	13 (76.5%)	17	0.12 (-0.11 to 0.34)
Total	0	2	4	0	23	38	
Participants without recent LBP							
Study visit 1							
Active SMT	0	4 (14.3%)	1 (3.6%)	0	23 (82.1%)	28	0.11 (-0.04 to 0.26)
Placebo SMT	1 (4.2%)	2 (8.3%)	0	0	21 (87.5%)	24	-0.13 (-0.26 to 0.01)
Total	1	6	1	0	44	52	
Study visit 2							
Active SMT	0	3 (11.1%)	2 (7.4%)	0	22 (81.5%)	27	0.04 (-0.12 to 0.20)
Placebo SMT	0	5 (20.8%)	0	0	19 (79.2%)	24	-0.21 (-0.37 to -0.05)
Total	0	8	2	0	41	51	

Section 4. Codebooks for qualitative thematic analysis

sCodebook 1. Categories, sub-categories, definitions and coded examples (participants)

Main category	Sub category	Sub category 2	Definition	Coded Examples
Sound	Audible release	—	Participants notice the presence or absence of an audible release (i.e., cavitation).	“It did not really crack”<br>“Because it cracked heavily and I feel freer in the chest area”<br>“Nothing cracked during the treatment”
Pain or discomfort	Pain or discomfort (decreased)	—	Participants report a decrease in pain or discomfort.	“Because the discomfort decreased”<br>“The pain in my mid back decreased”<br>“...and the pain decreased”
	Pain or discomfort (increased)	—	Participants express an increase in pain or discomfort.	“I don't know, but I have more pain in my lower back than before”<br>“Small improvement in flexibility, and mz [sic] back pain is more in pain now than before”<br>“I have slight discomfort in the back directly after the treatment”
Uncertainty	—	—	Participants report uncertainty (e.g., intervention assignment, intervention effect).	“No idea...just a feeling”<br>“I am not sure which treatment I received”<br>“Unsure if the treatment had any effect on the lumbar spine”
Experience	Experience with spinal manual therapy	—	Participants report previous experience receiving spinal manual therapy	“Corresponds to my idea of a regular chiropractic treatment”<br>“Because it didnt

			from various healthcare providers.	[sic] feel as traditional physiotherapy" "…like normal and gentle massage"
	No experience with spinal manual therapy	—	Participants lack experience with the spinal manual therapy intervention.	"I have no experience with chiropractic treatments,…" "No prior experience"
	Other experience	—	Participants describe familiarity/indirect experiences with spinal manual therapy.	"Felt familiar and the back felt more relaxed afterwards." "I've already seen it before with acquaintances."
**Comparison of intervention sessions**	Comparison of intervention sessions	—	Participants compare the second with the first intervention session.	"It was the same as last time, not much noticed." "Following the second treatment, I was sure that the first one was the control treatment." "The treatment felt less intense than the last time."
	Comparison of effects	—	Participants compare effects between the first and second intervention sessions.	"After the last treatment, a day later I felt much less pain in my lower back." "It was the same treatment as the last time, after that I had felt "nothing", so I think that's why it was the control." "Probably I had wrong impression the first time. This time I feel better and more flexible"
**Intervention**	Intervention simplicity	—	Participants discuss the simplicity of the intervention protocol.	"It seems quite simple and I do not see an immediate effect"

				"As mentioned before, the treatment seems quite simple"
	Intervention component	Prone mobilisation	Participants point to the prone mobilisation component of the intervention.	“Although unknown to me, I can not [sic] imagine that the bobbing [prone mobilisation] does any effect”<br>“...little stretching [prone mobilisation]...”
		Pressure or force (manual)	Participants account for the pressure or force of the intervention.	"Less pressure applied. Did not crack that much."<br>“There was no much pressure applied”<br>“the pushes were stronger”
		Breathing	Participants comment on the breathing intervention component.	“...not coupled with inhalation and exhalation”<br>“the pressure during exhalation made the treatment seem real”
		Duration	Participants refer to the duration of the intervention.	“I may be in the control group, since the treatment was short... The real treatment is probably longer.”<br>"...probably because it was [a] super short treatment."<br>"Length"
		Other	Participants point to other intervention components (e.g., intervention type, dose, intensity)	"Type of manipulation"<br>“Too little manual therapy”<br>"Slightly very gentle“ (adapted from German: ein bisschen sehr sanft)

	Intervention provider	Provider technique/execution	Participants comment on the intervention provider's technique or execution.	“Expert execution and explanation” “The doctor who performed the treatment palpated the back very precisely and the treatment technique seemed very real” "It felt like a real treatment due to the pressure and the doctors approach."
**General effects**	General effect (positive)	—	Participants perceive a general positive effect.	“I felt a soothing effect” “Because it felt quite good during the intervention and also afterwards” "I felt totally [sic] relaxed afterwards."
	General effect (marginal or none)	—	Participants experience a marginal or null effect.	"I did not feel any effect." "I do not feel any significant difference yet." "Because I didn't feel difference [sic] after the treatment."
	General effect (other)	—	Participants account for other general effects	—
**Musculoskeletal effect**	Musculoskeletal effect (positive)	—	Participants describe a positive musculoskeletal effect.	“My musculoskeletal system was readjusted” “It feels like the treatment has relieved the tension in the lower back” "Blockage in the upper back was dissolved."
	Musculoskeletal	—	Participants report	"Because I did not

	effect (marginal or none)		marginal or null musculoskeletal effects.	notice a big difference in back sensation between before and after the treatments."<br>"I also did not see any difference in my back function over a few days"
	Musculoskeletal effect (other)	—	Participants report other musculoskeletal effects.	"Because I felt pressure in the left back area after the treatment."
**Movement effect**	Movement (positive)	—	Participants report a positive effect on movement.	"Because mobility maximised and the pain decreased."<br>"I felt like in the post manipulation measurement I could bend backward a little more"<br>"Improved mobility of the lower back."
	Movement (marginal or none)	—	Participants point to a marginal or null movement effect.	"No cracking and no improvement in mobility."<br>"Because the mobility in the back has not changed extremely compared to before the treatment."<br>"Small improvement in flexibility..."
	Movement (other)		Participants comment on other movement effects.	—
**Impression**	Impression (general)		Participants discuss general impressions about the intervention.	“Because it was about measuring and light treatment”<br>"Treatment did not seem unusual"<br>"Felt coherent and dynamic."
**Expectations**		—	Participants describe their	“I expected to feel something in my

			intervention expectations.	back during the treatment, which was not the case”
**External factor**	Lifestyle factor	—	Participants report on an external lifestyle factor.	“It was good, but I have also been sitting less lately”
**Miscellaneous**	—	—	Participants point to their random intervention assignment or the randomisation component.	“Random” “Randomization”

sCodebook 2. Categories, sub-categories, definitions and coded examples (outcome assessors)

Main category	Sub category 1	Sub category 2	Definition	Coded examples
No change	No change (unspecific)	—	Outcome assessors notice no change in participants.	"No change"<br>"Participant looked the same"<br>"Patient looked the same"
Uncertainty	—	—	Outcome assessors report uncertainty.	"Not sure"<br>"No idea"
Movement	Range of motion	Perceived improved range of motion	Outcome assessors report an improvement in range of motion.	"I think a bit more rom"<br>"ROM was better"<br>"Much better ROM"
		Perceived marginal or no change in range of motion	Outcome assessors observe a marginal or no change in range of motion.	"ROM was the same"<br>"No ROM changes"<br>"Not much change in ROM"
	Movement quality	Perceived movement quality	Outcome assessors point to the movement quality of participants.	"Appears more smoothly"<br>"Not moving smooth"
Cues	Cues (unspecified)	—	Outcome assessors lack cues to inform their intervention assignment beliefs.	"No sign of anything"
	Cues (verbal)	—	Outcome assessors refer to participant statements to inform their intervention assignment beliefs.	"Did not talk"<br>"...and no verbal cues from the participant"<br>"Participant made a comment: Never experienced it soft like this"<br>"He said the chiro[practor] is a magician"<br>"Comments from the patient: This was great I feel like I can move way better, I will come again"
Participant expression	Perceived positive emotion	—	Outcome assessors perceive a positive participant emotion (e.g., gratification, relief of symptoms).	"The participant was a bit excited"<br>"Looked content"<br>"Patient felt reliefed [sic]"<br>"[The participant] seems a bit happier than before"
	Perceived indifference	—	Outcome assessors perceive indifference in participants.	"Did not look too impressed"<br>"Looked not impressed"

Section 5. Additional supplemental tables

sTable 12. Other participant outcomes

Outcome	Active SMT (n = 40)	Placebo SMT (n = 41)	Effect (95% CI)
Self-rated general health, No. (%)			
Study visit 1			
Fair/Poor	0 (0)	0 (0)	
Good	8 (20)	10 (24)	
Very good	23 (58)	23 (56)	
Excellent	9 (22.5)	8 (20)	
Study visit 2			
Fair/Poor	0 (0)	1 (2%)	
Good	9 (23)	16 (39)	
Very good	20 (51)	19 (46)	
Excellent	10 (26)	5 (12)	
Satisfaction with care, No. (%)			
Study visit 1			
Strongly agree/Agree	2 (5)	1 (2)	
Uncertain	5 (13)	9 (22)	
Disagree	9 (22)	12 (29)	
Strongly disagree	24 (60)	19 (46)	
Study visit 2			
Strongly agree/Agree	0 (0)	2 (5)	
Uncertain	2 (5)	6 (15)	
Disagree	9 (23)	5 (12)	
Strongly disagree	28 (72)	28 (68)	
Self-reported flexibility, No. (%)			
Study visit 1			
Very poor/Poor	2 (5)	2 (5)	
Average	13 (33)	13 (32)	
Good	15 (38)	12 (29)	
Very good	10 (25)	14 (34)	
Study visit 2			
Very poor/Poor	0 (0)	4 (10)	
Average	10 (26)	7 (17)	
Good	21 (54)	20 (49)	
Very good	8 (21)	10 (24)	
Pain intensity—Numeric Rating Scale, mean (SD)			
Change in mid back pain	-0.21 (0.95)	-0.07 (1.62)	-0.14 (-0.72 to 0.44)
Change in low back pain	-0.28 (1.67)	-0.10 (1.24)	-0.18 (-0.83 to 0.47)
Function— Numeric Rating Scale, mean (SD)			
Change in back function	0.38 (2.18)	-0.10 (1.48)	0.48 (-0.34 to 1.30)

sTable 13. Clinician-reported intervention delivery outcomes

Outcome	Active SMT (n = 40)	Placebo SMT (n = 41)
Participant tolerability of intervention		
Tolerated intervention protocol well, study visit 1, No. (%)	39 (98)	41 (100)
Tolerated intervention protocol well, study visit 2, No. (%)	39 (100)	40 (98)
Intervention component fidelity		
Side posture lumbar manipulation		
Delivered per protocol, study visit 1, No. (%)	40 (100)	41 (100)
Heard cavitation, study visit 1, No. (%)	38 (95)	1 (2)
Delivered per protocol, study visit 2, No. (%)	38 (97)	41 (100)
Heard cavitation, study visit 2, No. (%)	30 (77)	0
Prone lumbar mobilisation		
Delivered per protocol, study visit 1, No. (%)	40 (100)	41 (100)
Delivered per protocol, study visit 2, No. (%)	39 (100)	40 (98)
Prone thoracic manipulation		
Delivered per protocol, study visit 1, No. (%)	40 (100)	41 (100)
Heard cavitation, study visit 1, No. (%)	36 (90)	2 (5)
Delivered per protocol, study visit 2, No. (%)	39 (100)	41 (100)
Heard cavitation, study visit 2, No. (%)	33 (85)	3 (7)
Quality of intervention delivery relative to protocol		
Quality—study visit 1, Numeric Rating Scale, mean (SD)	8.72 (0.99)	8.39 (0.92)
Quality—study visit 2, Numeric Rating Scale, mean (SD)	8.79 (0.98)	8.56 (1.05)

sTable 14. Adverse events

	Active SMT (n = 40)	Placebo SMT (n = 41)
Any adverse event, No.	4	2
Musculoskeletal		
Increased pain or discomfort in the back	4	0
Vestibular		
Dizziness	0	2
Unique participants		
Unique participants with adverse events, No. (%)	3 (8)	1 (2)

Section 6. Blinding assessment tables for post hoc analyses

sTable 15. Blinding assessment by levels of gender (ITT)

	Beliefs						
Assignment	Strongly believe genuine	Somewhat believe genuine	Somewhat believe control	Strongly believe control	Do not know	Total	Bang BI
Women							
Study visit 1							
Active SMT	1 (6.7%)	8 (53.3%)	4 (26.7%)	0	2 (13.3%)	15	0.33 (-0.11 to 0.77)
Placebo SMT	1 (3.7%)	6 (22.2%)	10 (37.0%)	7 (25.9%)	3 (11.1%)	27	0.37 (0.04 to 0.70)
Total	2	14	14	7	5	42	
Study visit 2							
Active SMT	3 (20.0%)	6 (40.0%)	4 (26.7%)	0	2 (13.3%)	15	0.33 (-0.11 to 0.77)
Placebo SMT	0	8 (29.6%)	11 (40.7%)	7 (25.9%)	1 (3.7%)	27	0.37 (0.03 to 0.71)
Total	3	14	15	7	3	42	
Men							
Study visit 1							
Active SMT	3 (12.0%)	15 (60.0%)	2 (8.0%)	1 (4.0%)	4 (16.0%)	25	0.60 (0.33 to 0.87)
Placebo SMT	1 (7.1%)	3 (21.4%)	6 (42.9%)	3 (21.4%)	1 (7.1%)	14	0.36 (-0.11 to 0.83)
Total	4	18	8	4	5	39	
Study visit 2							
Active SMT	5 (20.8%)	11 (45.8%)	5 (20.8%)	2 (8.3%)	1 (4.2%)	24	0.38 (0.01 to 0.74)
Placebo SMT	0	5 (35.7%)	4 (28.6%)	3 (21.4%)	2 (14.3%)	14	0.14 (-0.34 to 0.62)
Total	5	16	9	5	3	38	

sTable 16. Blinding assessment by levels of intervention provider (ITT)

	Beliefs						
Assignment	Strongly believe genuine	Somewhat believe genuine	Somewhat believe control	Strongly believe control	Do not know	Total	Bang BI
Intervention provider 1—study visit 1							
Active SMT	1 (20.0%)	4 (80.0%)	0	0	0	5	1 (1 to 1)
Placebo SMT	0	2 (50.0%)	0	1 (25.0%)	1 (25.0%)	4	-0.25 (-1.06 to 0.56)
Total	1	6	0	1	1	9	
Intervention provider 1—study visit 2							
Active SMT	0	2 (100%)	0	0	0	2	1 (1 to 1)
Placebo SMT	0	2 (66.7%)	1 (33.3%)	0	0	3	-0.33 (-1.4 to 0.73)
Total		4	1	0	0	5	
Intervention provider 1—all visits							
Active SMT	1	6	0	0	0	7	1 (1 to 1)
Placebo SMT	0	4	1	1	1	7	-0.29 (-0.94 to 0.37)
Total	1	10	1	1	1	14	
Intervention provider 2—study visit 1							
Active SMT	1 (6.2%)	8 (50.0%)	4 (25.0%)	1 (6.3%)	2 (12.5%)	16	0.25 (-0.19 to 0.69)
Placebo SMT	0	3 (21.4%)	8 (57.1%)	2 (14.3%)	1 (7.1%)	14	0.50 (0.07 to 0.69)
Total	1	11	12	3	3	30	
Intervention provider 2—study visit 2							
Active SMT	2 (18.2%)	5 (45.5%)	1 (9.1%)	1 (9.1%)	2 (18.2%)	11	0.45 (-0.01 to 0.92)
Placebo SMT	0	2 (25.0%)	3 (37.5%)	3 (37.5%)	0	8	0.50 (-0.10 to 1.10)
Total	2	7	4	4	2	19	
Intervention provider 2—all visits							
Active SMT	3	13	5	2	4	27	0.33 (0.01 to 0.66)
Placebo SMT	0	5	11	5	1	22	0.50 (0.15 to 0.
Total	3	18	16	7	5	49	
Intervention provider 3—study visit 1							

Active SMT	1 (16.7%)	3 (50.0%)	0	0	2 (33.3%)	6	0.67 (0.29 to 1.04)
Placebo SMT	1 (20.0%)	1 (20.0%)	1 (20.0%)	2 (40.0%)	0	5	0.20 (-0.66 to 1.06)
Total	2	4	1	2	2	11	
Intervention provider 3—study visit 2							
Active SMT	1 (33.3%)	0	1 (33.3%)	1 (33.3%)	0	3	0.33 (-1.40 to 0.73)
Placebo SMT	0	2 (50.0%)	1 (25.0%)	1 (25.0%)	0	4	0 (-0.98 to 0.98)
Total	1	2	2	2	0	7	
Intervention provider 3—all study visits							
Active SMT	2	3	1	1	2	9	0.33 (-0.20 to 0.86)
Placebo SMT	1	3	2	3	0	9	0.11 (-0.54 to 0.76)
Total	3	6	3	4	2	18	
Intervention provider 4—study visit 1							
Active SMT	0	2 (50.0%)	1 (25.0%)	0	1 (25.0%)	4	0.25 (-0.56 to 1.06)
Placebo SMT	0	1 (12.5%)	4 (50.0%)	3 (37.5%)	0	8	0.75 (0.29 to 1.21)
Total	0	3	5	3	1	12	
Intervention provider 4—study visit 2							
Active SMT	1 (16.7%)	4 (66.7%)	1 (16.7%)	0	0	6	0.67 (0.07 to 1.26)
Placebo SMT	0	2 (50.0%)	1 (25.0%)	1 (25.0%)	0	4	0.00 (-0.98 to 0.98)
Total	1	6	2	1	0	10	
Intervention provider 4—all study visits							
Active SMT	1	6	2	0	1	10	0.50 (0 to 1)
Placebo SMT	0	3	5	4	0	12	0.5 (0.01 to 0.99)
Total	1	9	7	4	1	22	
Intervention provider 5—study visit 1							
Active SMT	1 (16.7%)	4 (66.7%)	1 (16.7%)	0	0	6	0.67 (0.07 to

							1.26)
Placebo SMT	0	0	1 (25.0%)	2 (50.0%)	1 (25.0%)	4	0.75 (0.33 to 1.17)
Total	1	4	2	2	1	10	
Intervention provider 5—study visit 2							
Active SMT	2 (33.3%)	2 (33.3%)	2 (33.3%)	0	0	6	0.33 (-0.42 to 1.09)
Placebo SMT	0	1 (16.7%)	2 (33.3%)	2 (33.3%)	1 (16.7%)	6	0.50 (-0.11 to 1.11)
Total	2	3	4	2	1	12	
Intervention provider 5—all study visits							
Active SMT	3	6	3	0	0	12	0.50 (0.01 to 0.99)
Placebo SMT	0	1	3	4	2	10	0.60 (0.19 to 1.01)
Total	3	7	6	4	2	22	
Intervention provider 6—study visit 1							
Active SMT	0	2 (66.7%)	0	0	1 (33.3%)	3	0.67 (0.13 to 1.20)
Placebo SMT	1 (16.7%)	2 (33.3%)	2 (33.3%)	0	1 (16.7%)	6	-0.17 (-0.88 to 0.55)
Total	1	4	2	0	2	9	
Intervention provider 6—study visit 2							
Active SMT	1 (25.0%)	1 (25.0%)	2 (50.0%)	0	0	4	0 (-0.98 to 0.98)
Placebo SMT	0	2 (33.3%)	2 (33.3%)	0	2 (33.3%)	6	0 (-0.65 to 0.65)
Total	1	3	4	0	2	10	
Intervention provider 6—all study visits							
Active SMT	1	3	2	0	1	6	0.29 (-0.37 to 0.94)
Placebo SMT	1	4	4	0	3	12	-0.08 (-0.57 to 0.40)
Total	2	7	6	0	4	18	
Intervention provider 7—study visit 1							
Active SMT	0	0	0	0	0	0	NA
Placebo SMT	0	0	0	0	0	0	NA
Total	0	0	0	0	0	0	

Intervention provider 7—study visit 2							
Active SMT	1 (14.3%)	3 (42.9%)	2 (28.6%)	0	1 (14.3%)	7	0.29 (-0.37 to 0.94)
Placebo SMT	0	2 (20.0%)	5 (50.0%)	3 (30.0%)	0	10	0.60 (0.10 to 1.1)
Total	1	5	7	3	1	17	
Intervention provider 7—all study visits							
Active SMT	1	3	2	0	1	7	0.29 (-0.37 to 0.94)
Placebo SMT	0	2	5	3	0	10	0.60 (0.10 to 1.1)
Total	1	5	7	3	1	17	
Intervention provider 8—all study visits							
Active SMT	0	0	0	0	0	0	NA
Placebo SMT	0	0	0	0	0	0	NA
Total	0	0	0	0	0	0	
Intervention provider 9—all study visits							
Active SMT	0	0	0	0	0	0	NA
Placebo SMT	0	0	0	0	0	0	NA
Total	0	0	0	0	0	0	

sTable 17. Blinding assessment of participants without any protocol deviation (sensitivity)

	Beliefs						
Assignment	Strongly believe genuine	Somewhat believe genuine	Somewhat believe control	Strongly believe control	Do not know	Total	Bang BI
Study visit 1							
Active SMT	2 (5.7%)	22 (62.9%)	5 (14.3%)	1 (2.9%)	5 (14.3%)	35	0.51 (0.26 to 0.77)
Placebo SMT	2 (5.4%)	7 (18.9%)	16 (43.2%)	8 (21.6%)	4 (10.8%)	37	0.41 (0.13 to 0.68)
Total	4	29	21	9	9	72	
Study visit 2							
Active SMT	6 (17.6%)	16 (47.1%)	7 (20.6%)	2 (5.9%)	3 (8.8%)	34	0.38 (0.09 to 0.68)
Placebo SMT	0	12 (32.4%)	14 (37.8%)	8 (21.6%)	3 (8.1%)	37	0.27 (-0.03 to 0.57)
Total	6	28	21	10	6	71	
